# Adenosine Receptor Stimulation by Polydeoxyribonucleotide Improves Tissue Repair and Symptomology in Experimental Colitis

**DOI:** 10.3389/fphar.2016.00273

**Published:** 2016-08-23

**Authors:** Giovanni Pallio, Alessandra Bitto, Gabriele Pizzino, Federica Galfo, Natasha Irrera, Francesco Squadrito, Giovanni Squadrito, Socrate Pallio, Giuseppe P. Anastasi, Giuseppina Cutroneo, Antonio Macrì, Domenica Altavilla

**Affiliations:** ^1^Section of Pharmacology, Department of Clinical and Experimental Medicine, Medical School, University of Messina Messina, Italy; ^2^Department of Human Pathology, University of Messina Messina, Italy; ^3^Department of Biomedical Sciences and Morphological and Functional Images, University of Messina Messina, Italy

**Keywords:** colitis, A_2A_ agonist, PDRN, inflammation, apoptosis

## Abstract

Activation of the adenosine receptor pathway has been demonstrated to be effective in improving tissue remodeling and blunting the inflammatory response. Active colitis is characterized by an intense inflammatory reaction resulting in extensive tissue damage. Symptomatic improvement requires both control of the inflammatory process and repair and remodeling of damaged tissues. We investigated the ability of an A_2A_ receptor agonist, polydeoxyribonucleotide (PDRN), to restore tissue structural integrity in two experimental colitis models using male Sprague-Dawley rats. In the first model, colitis was induced with a single intra-colonic instillation of dinitrobenzenesulfonic acid (DNBS), 25 mg diluted in 0.8 ml 50% ethanol. After 6 h, animals were randomized to receive either PDRN (8 mg/kg/i.p.), or PDRN + the A_2A_ antagonist [3,7-dimethyl-1-propargylxanthine (DMPX); 10 mg/kg/i.p.], or vehicle (0.8 ml saline solution) daily. In the second model, dextran sulfate sodium (DSS) was dissolved in drinking water at a concentration of 8%. Control animals received standard drinking water. After 24 h animals were randomized to receive PDRN or PDRN+DMPX as described above. Rats were sacrificed 7 days after receiving DNBS or 5 days after DSS. In both experimental models of colitis, PDRN ameliorated the clinical symptoms and weight loss associated with disease as well as promoted the histological repair of damaged tissues. Moreover, PDRN reduced expression of inflammatory cytokines, myeloperoxidase activity, and malondialdehyde. All these effects were abolished by the concomitant administration of the A_2A_ antagonist DMPX. Our study suggests that PDRN may represent a promising treatment for improving tissue repair during inflammatory bowel diseases.

## Introduction

Inflammatory bowel diseases (IBDs) such as Crohn’s disease and ulcerative colitis are chronic diseases of the gastrointestinal tract characterized by inflammation of the enteric wall, abdominal pain, diarrhea, bleeding, and malabsorption. The etiology of these diseases remain unknown, although increasing evidence suggests that IBDs arise from altered immunological, genetic, and environmental factors (Podolsky, 2002). The mucosal and submucosal alterations characteristically observed in IBDs are deep, longitudinal ulcerations, accompanied by hemorrhage, submucosal edema and neutrophil infiltration. When IBDs become chronic the colon becomes a rigid foreshortened tube that lacks its usual haustral markings. These alterations are due, at least in part, to the presence of pro-inflammatory cells, free radicals, cytokines, eicosanoids, and chemotactic factors (Pavlick et al., 2002; Rahimian et al., 2010). During inflammation, the activation of nuclear factor-κB (NF-κB) causes adaptive modifications in the damaged cells, promoting the further production of pro-inflammatory mediators that enlarge the extent of the damaged tissue (Shaulian and Karin, 2012). Specifically, activation of NF-κB increases tumor necrosis factor-alpha (TNF-α) expression, which can induce either apoptosis or necrosis depending on the targeted cell type, environmental conditions, and magnitude of the cellular insult. In addition, the death receptors tumor necrosis factor receptor superfamily member 6 (FAS), tumor necrosis factor receptor 2 (TNFR2), TNF-related apoptosis-inducing ligand receptor 1 (TRAILR1), and TNF-related apoptosis-inducing ligand receptor 2 (TRAILR2), which are characteristically associated with apoptosis, might also induce necrosis after caspase blockage or starvation (Nunes et al., 2014).

Conventional therapies for IBD include anti-inflammatory drugs, such as aminosalicylates, corticosteroids, thiopurines, methotrexate, and anti-tumor necrosis factor agents (Burger and Travis, 2011). The long-term use of these drugs can induce severe side effects with a negative impact on patients’ quality of life (Baumgart and Sandborn, 2007). For this reason, there is a need for new, effective therapies with fewer side effects. Previous studies have indicated that adenosine receptor stimulation may down-regulate inflammation (Odashima et al., 2005). The adenosine receptors (A1, A_2A_, A_2B_, and A_3_) are expressed on immune/inflammatory cells, such as lymphocytes, neutrophils, monocytes, and macrophages (Fortin et al., 2006; Desrosiers et al., 2007). Moreover, it has been shown that stimulation of A_2A_ reduces pro-inflammatory cytokines in different disease models, including asthma, arthritis, and sepsis (Haskó et al., 2008).

Our previous work (Bitto et al., 2011, 2013), has characterized the anti-inflammatory and tissue repair activity of a specific A_2A_ receptor agonist, polydeoxyribonucleotide (PDRN) which is extracted from trout (grown in aquaculture for feeding purposes) with a registered method (Registration Dossier, Italian Ministry of Health; Rome, Italy). The purified PDRN contains a mixture of polynucleotides, without contaminating pharmacologically active proteins and peptides (data on file). The drug is already on the market, therefore the eventually positive effects of this agent may be promptly translated into clinics. PDRN has been shown to improve tissue repair in animal models (Galeano et al., 2008; Bitto et al., 2011) and has demonstrated efficacy in a recent clinical trial (Squadrito et al., 2014). The purpose of this study was to investigate the effects of PDRN in improving tissue remodeling and reducing the inflammatory response during experimental inflammatory colitis induced with either 2,4-dinitrobenzenesulfonic acid (DNBS) or dextran sulfate sodium (DSS) which are the most commonly used models for human colitis.

## Materials and Methods

### Animals and Drugs

All animal procedures were in accordance with the Principles of Laboratory Animal Care (NIH publication no. 85-23, revised 1985), authorized by our Local Institution (protocol #03/2013), and were in accordance with the ARRIVE Guidelines (McGrath et al., 2010). A total of 56 male Sprague-Dawley rats (14 weeks old, 250–300 g) were purchased from Charles River Laboratories (Calco, Milan, Italy). Animals were maintained in plastic cages under standard environmental conditions and feed *ad libitum* in the Animal Facility of the Department of Clinical and Experimental Medicine of the University of Messina, Messina, Italy. Animals were allowed to acclimatize for 1 week before the beginning of the experiments.

PDRN was a kind gift of Mastelli S.R.L. (Sanremo, Italy) and was prepared fresh daily in a 0.9% NaCl solution. The dose and route of administration were chosen according to previously published reports (Galeano et al., 2008; Altavilla et al., 2011; Bitto et al., 2011; Polito et al., 2012). DNBS was purchased from Sigma-Aldrich (Milan, Italy) and dissolved in 50% ethanol. DSS (MW = 36,000–50,000), was purchased from MP Biomedicals (Solon, OH, USA) and prepared daily in fresh drinking water at 8%. 3,7-Dimethyl-1-propargylxanthine (DMPX), an antagonist of A_2A_ receptor, was purchased from Sigma-Aldrich and was prepared fresh daily in dimethyl sulfoxide (DMSO) and then 1:10 in saline. The dose and route of administration were chosen according to previously published reports (Munzar et al., 2002; Arolfo et al., 2004; Yao et al., 2006; Thorsell et al., 2007; Balasubramanyan and Sharma, 2008)

### DNBS Model

Colitis was induced in 21 fasted rats under light anesthesia by a single intra-colonic instillation of DNBS (25 mg in 0.8 ml 50% ethanol) through a catheter that was inserted into the colon (for 8 cm) via the anus up to the splenic flexure. Animals were then kept for 15 min in Trendelenburg position to avoid reflux and after 6 h were randomized to receive the vehicle (1 ml/kg; *n* = 7), PDRN (8 mg/kg, *n* = 7), or PDRN+DMPX; (10 mg/kg; *n* = 7) intraperitoneally. The treatment was administered daily until the day of sacrifice. Control animals (*n* = 7) received a single intra-colonic instillation of 0.8 ml saline solution. At day 7, rats were sacrificed, the abdomen was opened by a midline incision and the descending colon was removed, opened along the anti-mesenteric border, rinsed and cut into two equal pieces, one stored for histological assessments, the other for measurement of biochemical markers. Blood was collected by heart puncture, centrifuged, and kept frozen until analysis.

### DSS Model

Colitis was induced in 21 rats by oral administration of 8% DSS dissolved in drinking water from day 0 to day 5, as reported by Onishi et al. (2015). After 24 h, the animals were randomized to receive either PDRN (*n* = 7), or PDRN+DMPX (*n* = 7), or vehicle (*n* = 7) as above for the duration of the study. Control animals (*n* = 7) received standard drinking water. At day 5, animals were sacrificed, blood and colon samples were collected and stored as described above.

### Clinical Assessment of Colitis

In both models the body weight, food intake, stool consistency, rectal bleeding, or the presence of blood in the stool were monitored daily for the duration of the study. Body weight and food intake were recorded every day between 9:00 and 10:00 a.m. from the day of colitis induction to the end of the experiment. Body weight results were expressed as raw data and compared with food intake, by using the following formula: food intake divided by body weight in grams and multiplied by 100. In the DSS model water consumption, from a graduated bottle was recorded every day between 9:00 and 10:00 a.m. from day 0 to the end of the experiment.

### Macroscopic Damage Score

Colon length was measured and damage was scored by two independent observers, as previously described (Wallace et al., 1992), according to the following criteria: 0 (no damage), 1 (localized hyperemia without ulcers), 2 (linear ulcers with no significant inflammation), 3 (linear ulcers with inflammation at one site), 4 (two or more major sites of inflammation and ulceration extending 1 cm along the length of the colon), and 5–8 (one point is added for each centimeter of ulceration beyond an initial 2 cm).

### Microscopic Damage Score

For light microscopy, colon tissues were fixed in 10% buffered formalin for 24 h, then specimens were embedded in paraffin, sectioned at 5 μm thickness, stained with hematoxylin and eosin (H&E), and observed with a Leica microscope (Leica Microsystems, Milan, Italy). Assessment of tissue changes was carried out by two observers blinded to the experimental protocol. The following morphological criteria were considered: score 0 (no damage), score 1 (mild: focal epithelial edema and necrosis), score 2 (moderate: diffuse swelling and necrosis of the goblet cells), score 3 (severe: necrosis and neutrophil infiltrate in the submucosa), score 4 (very severe: widespread necrosis with massive neutrophil infiltrate and hemorrhage) as previously reported (Rahimian et al., 2010).

### Immunohistochemical Evaluation of Bax and Bcl-2

Paraffin-embedded tissues were sectioned (5 μm), rehydrated, and antigen retrieval was performed by using 0.05 M sodium citrate buffer (pH 6.0) in a microwave for 5 min. Tissues were treated with 1% hydrogen peroxide to block endogenous peroxidase activity, and with normal horse serum (Vector Laboratories, Burlingame, CA, USA) to prevent non-specific staining. Primary antibodies against either Bax (Abcam, Cambridge, UK) or Bcl-2 (Cell Signaling Technologies Danvers, MA, USA) were used and the slides were kept overnight at 4°C in a humid box. The slides were then washed in phosphate buffer sodium (PBS), the appropriate secondary antibody was added, and the ABC system (Vectastain Elite ABC kit, Vector Laboratories) was used to detect antibody localization. The location of the reaction was visualized with diaminobenzidine tetra-hydrochloride (Sigma-Aldrich, Milan, Italy). Slides were counterstained with hematoxylin, dehydrated, and mounted with coverslips. As a part of the histologic evaluation, all slides were de-identified with regards to treatment group and evaluated by a pathologist at 5× to 40× magnification with a Leica microscope (Leica Microsystems, Milan, Italy).

### Measurement of Myeloperoxidase Activity

Myeloperoxidase (MPO), a marker of polymorphonuclear leukocyte accumulation, was determined as previously described (Mullane et al., 1985). Equal amounts of colon tissue were homogenized mechanically with the MICCRA D-1 homogenizer (Miccra Gmbh, Müllheim, Germany), in a solution containing 0.5% hexadecyltrimethylammonium bromide dissolved in 10 mM potassium phosphate buffer (pH 7.0). Lysates were then centrifuged for 30 min at 15,000 rpm at 4°C. An aliquot of the supernatant was allowed to react with a solution of 1.6 mM tetra-methyl-benzidine and 0.1 mM H_2_O_2_. The absorbance was measured with a spectrophotometer at 650 nm. MPO activity was defined as the quantity of enzyme degrading 1 μmol hydrogen peroxide/min at 37°C and was expressed in units per gram of tissue.

### Malondialdehyde Measurement

The levels of malondialdehyde (MDA) in the colon were determined as an indicator of lipid peroxidation (Ohkawa et al., 1979). Equal amounts of colon tissue were homogenized mechanically with the MICCRA D-1 homogenizer (Miccra Gmbh, Müllheim, Germany), in 1.15% KCl solution, a 0.1 ml aliquot of the homogenate was added to a reaction mixture containing 0.2 ml of 8.1% sodium dodecyl sulfate (SDS), 1.5 ml of 20% acetic acid, 1.5 ml of 0.8% thiobarbituric acid and 700 ml distilled water. Samples were boiled for 1 h at 95°C and centrifuged at 3000×*g* for 10 min. The absorbance of the supernatant was measured by spectrophotometer at 650 nm.

### Evaluation of IL-1β and TNF-α

Serum samples were assayed in duplicate using commercially available enzyme linked immunosorbent assay (ELISA) kits for Interleukin 1 beta (IL-1β) and TNF-α (Abcam, Cambridge, UK). The absorbance was read at 405 nm and the results were obtained interpolating the measurements with the respective standard curves. For each sample, the mean of the duplicates was used and expressed in pg/ml.

### Statistical Analysis

All quantitative data are expressed as mean ± SEM for each group and compared by one-way or two-way ANOVA for non-parametric variables with Tukey’s post-test for intergroup comparisons. Statistical significance was set at *p* < 0.05. Graphs were drawn using GraphPad Prism software version 5.0 for Windows (GraphPad Software Inc., La Jolla, CA, USA).

## Results

### Effects of PDRN on Clinical Signs

**FIGURE 1:**
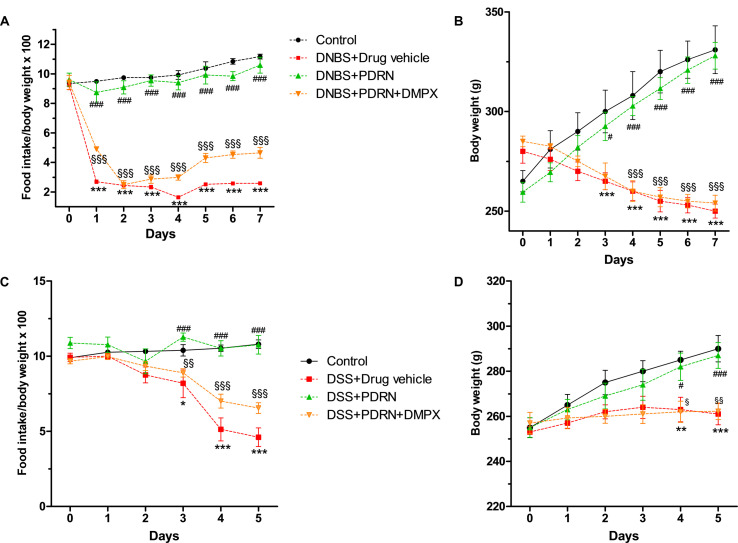
**Clinical evaluations.** Food intake in DNBS-treated animals **(A)**, weight loss in DNBS-treated animals **(B)**, food intake in DSS-treated animals **(C)**, and weight loss in DSS-treated animals **(D)** was recorded. Values were obtained from seven animals per group and are expressed as means and SEM. ^∗^*p* < 0.01, ^∗∗^*p* < 0.001, ^∗∗∗^*p* < 0.0001 vs control group; ^#^*p* < 0.01, ^###^*p* < 0.0001 vs DNBS/DSS+drug vehicle group; ^§^ *p* < 0.01, ^§§^ *p* < 0.001, ^§§§^ *p* < 0.0001 vs DNBS/DSS+PDRN group.

Colitis was successfully induced in rats as early as 6 h after DNBS treatment as demonstrated by the appearance of diarrhea followed by significant loss in body weight from day 3. PDRN-treated animals demonstrated an initial slight weight reduction (not significant) followed by a recovery by the end of the experiment (*p* < 0.0001 vs animals treated with DNBS+drug vehicle) with the appearance of solid feces after 3 days (**Figures 1A,B**). The concomitant administration of DMPX prevented weight and appetite loss in the DNBS+PDRN+DMPX group. Furthermore, at the end of the study, this group still had diarrhea, suggesting that DMPX completely blocked the effectiveness of PDRN in these animals (**Figures 1A,B**).

DSS-administered animals showed diarrhea and rectal bleeding after 24 h, while weight reduction was observed starting from day 3 (*p* < 0.0001 vs control group at the end of experiment). Treatment with PDRN resulted in reduced bleeding and improved food intake and body weight (*p* < 0.0001 vs DSS+drug vehicle at the end of the experiment; **Figures 1C,D**). DMPX abolished the positive effect of PDRN on food intake and body weight in DSS administered animals. Rectal bleeding was still present in these animals at the end of the study, indicating that DMPX fully antagonized the effects of PDRN (**Figures 1C,D**).

### Effects of PDRN on Macroscopic Damage

**FIGURE 2:**
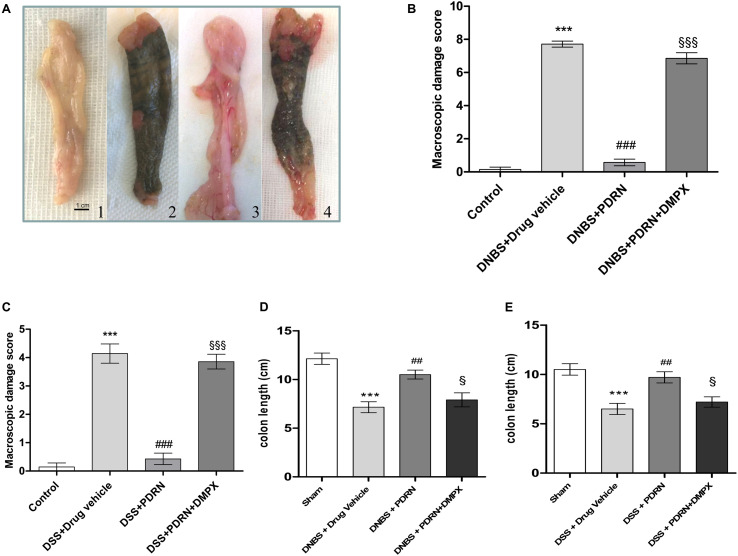
**Macroscopic evaluations.** Colons **(A)** from control **(1)**, DNBS+drug vehicle **(2)**, DNBS+PDRN **(3)**, DNBS+PDRN+DMPX **(4)** are depicted. Macroscopic damage scores from the DNBS model and the DSS model are summarized in **(B)** and **(C)**, respectively, while colon length is shown in **(D)** and **(E)** for these models. Values were obtained from seven animals per group, and are expressed as the means and SEM. ^∗∗∗^*p* < 0.0001 vs control group; ^##^*p* < 0.001, ^###^*p* < 0.0001 vs DNBS/DSS+drug vehicle group; ^§^ *p* < 0.01, ^§§§^ *p* < 0.0001 vs DNBS/DSS+PDRN group.

At the end of the experiment, the colon of animals in the DNBS+drug vehicle group appeared ulcerated, edematous, and hyperemic compared to control group (*p* < 0.0001; **Figures 2A,B**). In the group treated with PDRN a significant reduction in the extent and severity of colon injury was observed (*p* < 0.0001 vs DNBS+drug vehicle group; **Figures 2A,B**). As shown in **Figure 2D**, DNBS also caused a significant shortening of the colon, as compared to control animals (*p* < 0.0001). Treatment with PDRN significantly improved this condition. The positive effects of PDRN administration on macroscopic damage were blunted by DMPX (**Figures 2A–D**).

In the DSS+drug vehicle group, as well as in the DSS+PDRN+DMPX group, the presence of non-formed feces and blood was observed at the end of the study. However, the animals receiving PDRN demonstrated normal colon appearance in contrast to the animals that did not receive PDRN (**Figure 2C**). As shown in **Figure 2E**, DSS caused a significant reduction in colonic length as compared to control animals (*p* < 0.0001). Treatment with PDRN significantly improved this condition. No difference was observed in water consumption among groups during the study period.

### PDRN Reduces Histological Damage

**FIGURE 3:**
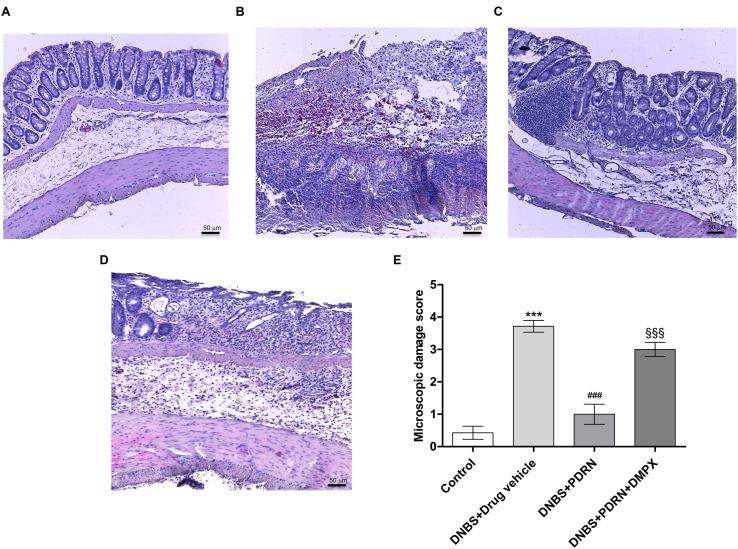
**Histological evaluations from DNBS-treated animals.** Representative photomicrographs of H&E stained tissues (original magnification 10×) derived from control **(A)**, DNBS+drug vehicle **(B)**, DNBS+PDRN **(C)**, or DNBS+PDRN+DMPX **(D)** animals are shown. The graph represents the microscopic damage score **(E)**, where values are expressed as the means and SEM of seven animals. ^∗∗∗^*p* < 0.0001 vs control group; ^###^*p* < 0.0001 vs DNBS+drug vehicle group; ^§§§^*p* < 0.0001 vs DNBS+PDRN group. Scale bar 50 μm.

A normal appearance of the colonic mucosa with intact epithelium was observed in the control group (**Figures 3A,E**). DNBS administration resulted in epithelial necrosis, massive infiltration of neutrophils and macrophages into the mucosal and submucosal layers, thickening of the colon wall, loss of goblet cells and edema (**Figures 3B,E**). The administration of PDRN resulted in: (i) a significant reduction in the extent and severity of epithelial and mucosal alterations; (ii) regeneration of the epithelium with preservation of crypts and goblet cells; and (iii) reduced infiltration of inflammatory cells (**Figures 3C,E**). Blockade of the adenosine receptor with DMPX counteracted the beneficial effects of PDRN (**Figures 3D,E**).

**FIGURE 4:**
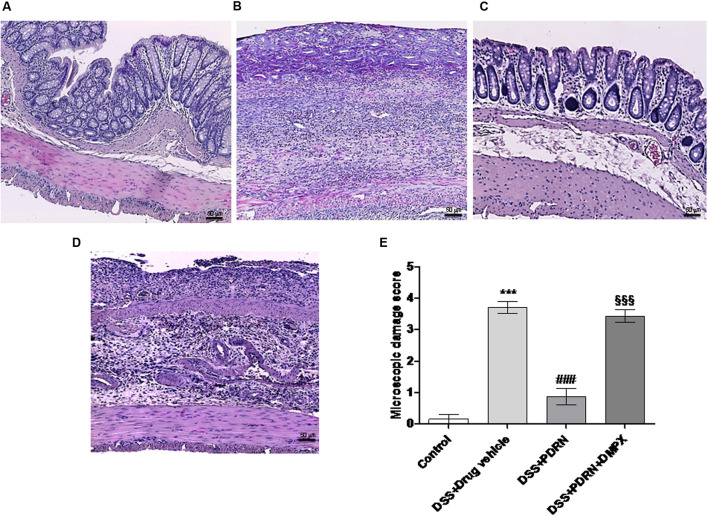
**Histological evaluations from DSS-treated animals.** Representative H&E images (original magnification 10×) of tissues derived from control **(A)**, DSS+drug vehicle **(B)**, DSS+PDRN **(C)**, or DSS+PDRN+DMPX **(D)** treated animals are shown here. The graph in **(E)** represents the microscopic damage score values expressed as means and SEM derived from seven animals for each condition. ^∗∗∗^*p* < 0.0001 vs control group; ^###^*p* < 0.0001 vs DSS+drug vehicle group; ^§§§^ *p* < 0.0001 vs DSS+PDRN group. Scale bar 50 μm.

In the DSS model, the histological analysis showed a flattening of the mucosa with a reduced presence of mucosal glands, a massive inflammatory infiltrate and edema in the submucosal layer (**Figures 4B,E**) as compared to control animals (**Figures 4A,E**). Treatment with PDRN significantly reduced the extent and severity of the histological alteration associated with DSS administration and stimulated regeneration of the epithelium, crypts, and goblet cells (**Figures 4C,E**). DMPX administration abrogated the protective effects of PDRN on histological features (**Figures 4D,E**).

### Effects of PDRN on Apoptosis Markers

**FIGURE 5:**
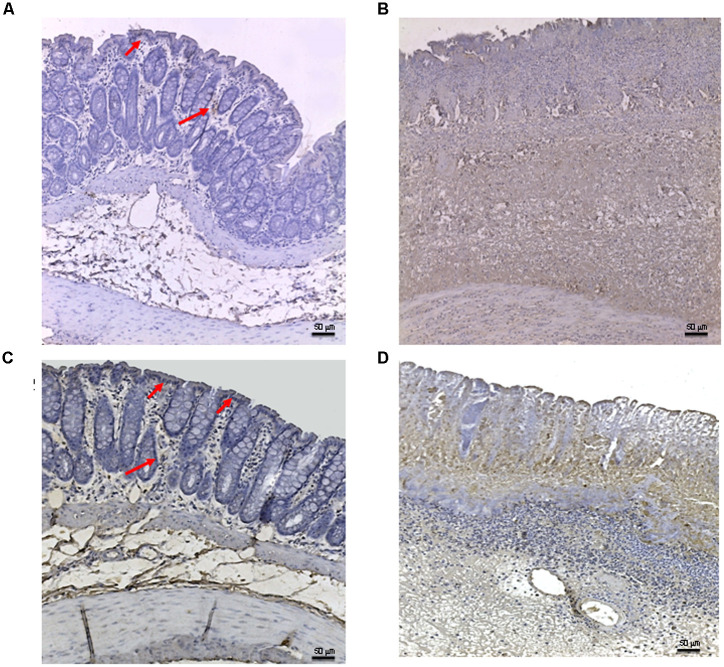
**Bax immunostaining from DNBS-treated animals.** Representative Bax immunostaining (original magnification 10×) of colons derived from control **(A)**, DNBS+drug vehicle **(B)**, DNBS+PDRN **(C)**, or DNBS+PDRN+DMPX **(D)** treated animals are depicted. The arrows in **(A)** and **(C)** point to areas of slight positivity. The pictures in **(B,D)** demonstrate a diffuse staining for the apoptotic factor. Scale bar 50 μm.

**FIGURE 6:**
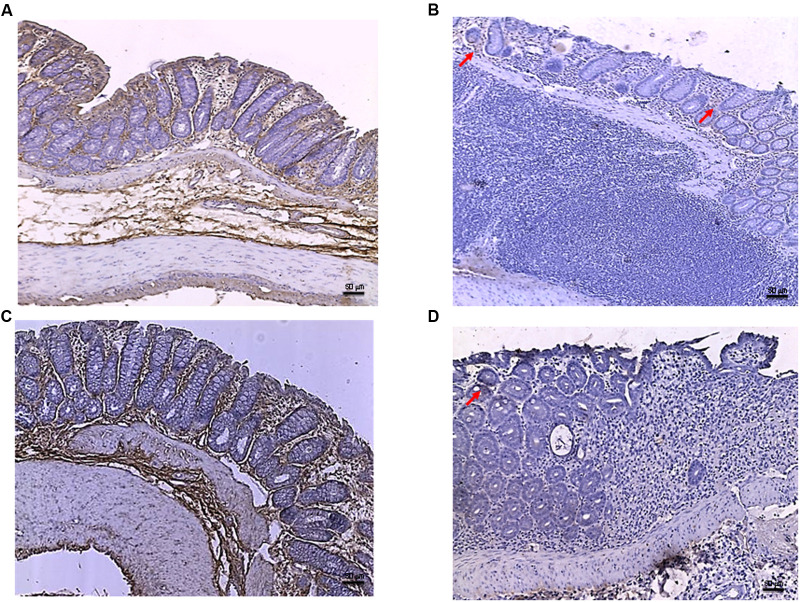
**Bcl-2 immunostaining from DNBS-treated animals.** Representative Bcl-2 immunostaining (original magnification 10×) of tissues derived from control **(A)**, DNBS+drug vehicle **(B)**, DNBS+PDRN **(C)**, or DNBS+PDRN+DMPX **(D)** treated animals are depicted. The images in **(A,C)** demonstrate diffuse Bcl-2 staining, while the arrows in **(B,D)** point to the residual Bcl-2 staining in the mucosal layer. Scale bar 50 μm.

Tissues taken from control rats of both experimental models demonstrated, as expected, very little staining for Bax (**Figure 5A**). Sections obtained from the DNBS+drug vehicle group exhibited a diffuse positive staining for Bax (**Figure 5B**), demonstrating the activation of apoptosis in the epithelial and submucosal layers. PDRN administration markedly reduced the degree of Bax positive staining, almost restoring a normal appearance of the colon (**Figure 5C**), while DMPX co-administration abrogated the protective effect of PDRN (**Figure 5D**). The Bcl-2 staining in the control group from both experiments was highly positive (**Figures 6A** and **8A**). DNBS administration almost abrogated the presence of Bcl-2 due to the massive necrosis of the tissue (**Figure 6B**). PDRN treatment restored the presence of Bcl-2 (**Figure 6C**) in the newly formed mucosal layer, however, when co-administered with DMPX the beneficial effects were no longer observed (**Figure 6D**).

**FIGURE 7:**
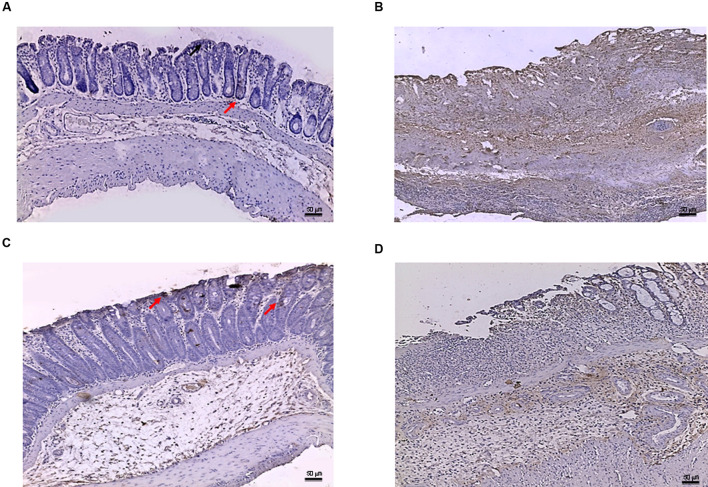
**Bax immunostaining from DSS-treated animals.** Representative Bax immunostaining (original magnification 10×) of colons derived from control **(A)**, DSS+drug vehicle **(B)**, DSS+PDRN **(C)**, or DSS+PDRN+DMPX **(D)** treated animals are shown. The arrows in **(A,C)** point to regions of slight positivity. The pictures in **(B,D)** demonstrate a diffuse staining for the apoptotic factor. Scale bar 50 μm.

**FIGURE 8:**
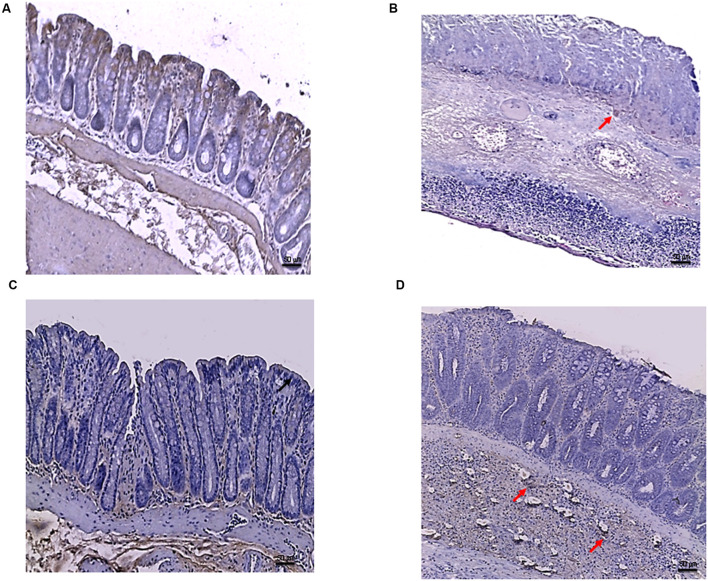
**Bcl-2 immunostaining from DSS-treated animals.** Representative images of Bcl-2 immunostaining (original magnification 10×) of colons derived from control **(A)**, DSS+drug vehicle **(B)**, DSS+PDRN **(C)**, or DSS+PDRN+DMPX **(D)** treated animals are shown. The image in **(A,C)** demonstrate diffuse staining for Bcl-2, while the arrows in **(B,D)** point to residual Bcl-2 staining in the mucosal layer. Scale bar 50 μm.

In the DSS model, the immunostaining for Bax revealed wide expression of the pro-apoptotic molecule in DSS animals compared to controls (**Figures 7A,B**) that was reduced to basal levels by PDRN treatment (**Figure 7C**). A strong reduction in Bcl-2 staining was observed in the DSS+drug vehicle group (**Figure 8B**). The administration of PDRN was able to restore Bcl-2 positivity in the regenerated tissue (**Figure 8C**). The co-administration of DMPX blunted the effects of PDRN on Bax and Bcl-2 expression (**Figures 7D** and **8D**).

### Effects of PDRN on Lipid Peroxidation and Neutrophil Infiltration

**FIGURE 9:**
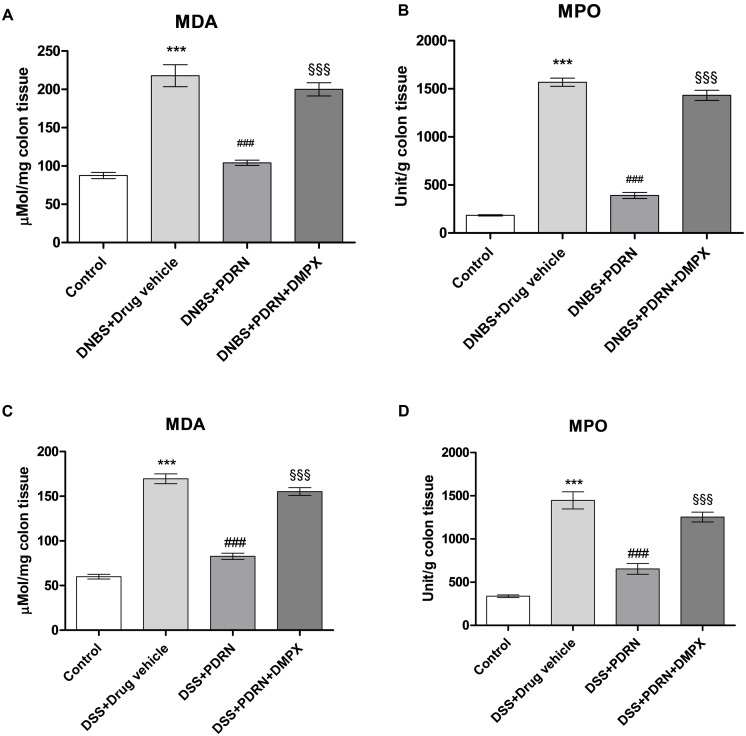
**Evaluation of inflammatory markers.** The effects of PDRN on malondialdehyde levels and myeloperoxidase activity in DNBS-treated animals are shown in **(A)** and **(B)**, respectively, while the consequences of PDRN treatment on malondialdehyde levels and myeloperoxidase activity in the DSS model are summarized in **(C)** and **(D)**, respectively. All values are expressed as means and SEM based on observations made on seven animals per group. ^∗∗∗^*p* < 0.0001 vs control group; ^###^*p* < 0.0001 vs DNBS/DSS+drug vehicle group; ^§§§^ *p* < 0.0001 vs DNBS/DSS+PDRN group.

MDA levels and MPO activity were increased in the groups of animals that received DNBS+drug vehicle and DSS+drug vehicle (**Figures 9A–D**). The administration of PDRN markedly reduced both lipid peroxidation and the accumulation of polymorphonuclear granulocytes, in both experimental models (**Figures 9A–D**). DMPX, abolished the protective effects of PDRN on both MDA level and MPO activity (**Figures 9A–D**).

### Effects of PDRN on IL-1 β and TNF-α levels

**FIGURE 10:**
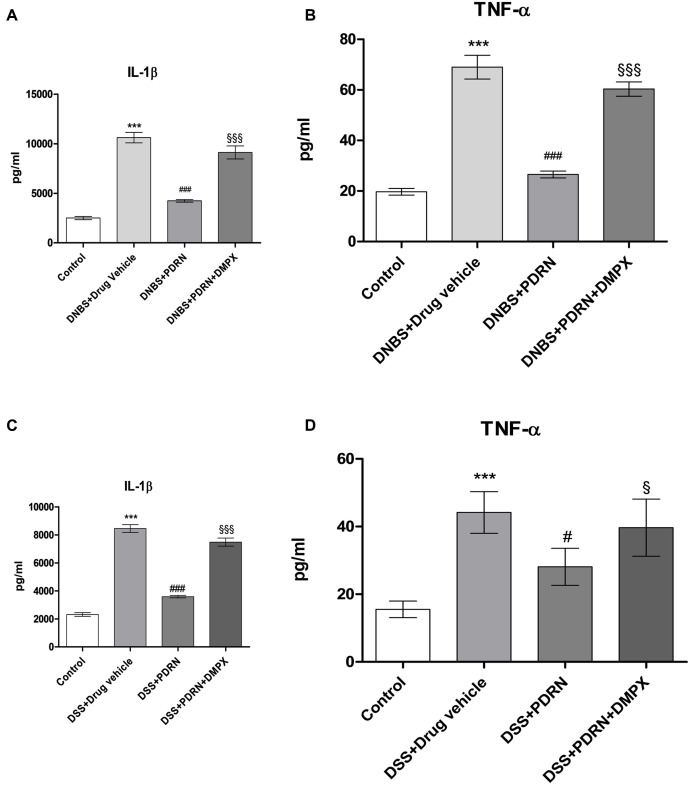
**Evaluation of inflammatory cytokines.** Serum of DNBS- or DSS-challenged animals was extracted, and Interleukin 1β (IL-1β) **(A,C)** and tumor necrosis factor-alpha (TNF-α) **(B,D)** levels were determined by enzyme linked immunosorbent assay (ELISA). Values shown here are expressed as the means and SEM of seven animals per group. ^∗∗∗^*p* < 0.0001 vs control group; ^#^*p* < 0.01, ^###^*p* < 0.0001 vs DNBS/DSS+drug vehicle group; ^§^ *p* < 0.01, ^§§§^ *p* < 0.0001 vs DNBS/DSS+PDRN group.

Circulating levels of the proinflammatory cytokines IL-1β and TNF-α were measured 7 days after DNBS administration. As shown in **Figures 10A,B**, a significant increase in both IL-1β and TNF-α was found in serum from DNBS+drug vehicle group compared to control animals (*p* < 0.0001). A_2A_ stimulation reduced the serum levels of IL-1β and TNF-α significantly (*p* < 0.0001 vs DNBS+drug vehicle group). In the DSS model, IL-1β and TNF-α were similarly upregulated (**Figures 10C,D**), and PDRN reduced their expression. Again, PDRN failed to elicit an inhibitory effect on cytokine levels in the presence of DMPX (**Figures 10A–D**).

## Discussion

Despite the unknown etiology of IBDs, oxidative stress appears to have an important role in the development of epithelial damage, altered immune response, and increased cytokine production (Rachmilewitz et al., 1993; Grisham, 1994; Strober and Fuss, 2011). The intracellular pathways triggered by adenosine receptor stimulation have a well-recognized role in modulating the inflammatory cascade in the gastro-intestinal tract and elsewhere (Antonioli et al., 2010). All four adenosine receptors, A1, A_2A_, A_2B_, and A3, are expressed in colonic epithelial cells. In addition, A1 receptors were found to be expressed in circular muscle, while A_2A_ receptors were detected in both circular muscle and myenteric plexus (Barrett et al., 1989; Fornai et al., 2009; Lam et al., 2009). It has been previously reported that the activation of A_2A_ modulates the inflammatory response and stimulates epithelial repair in experimental colitis (Antonioli et al., 2011, 2014). Moreover, when colitis was induced in A_2A_ knockout mice via *Clostridium difficile* infection, knockout animals had a worse outcome compared to wild type littermates (Li et al., 2012), further supporting a role for A_2A_ receptor in colitis. In addition, A_2B_ stimulation has recently been shown to have a protective role in colitis (Aherne et al., 2015) and protects the colonic epithelial barrier during acute colitis via enhancing mucosal barrier responses.

Previous findings in several mouse models of inflammatory and immune disease (Haskó et al., 2008) and in other animal models of IBD (Odashima et al., 2005; Cavalcante et al., 2006) have demonstrated the efficacy of A_2A_ receptor stimulation. Therefore, it is not surprising that PDRN restores the enteric mucosa in these colitis models. Although it seems unlikely, due to different route of administration and mechanism of action, it should be acknowledged that PDRN could mitigate symptoms by interfering with the damaging agents (DNBS and DSS). On the other hand, it has been suggested that adenosine generated by the catabolizing enzyme CD73 during bacterial infection can contribute to immunosuppression leading to increased pathogen load (Alam et al., 2015). Our present data do not support an increase in tissue damage due to adenosine receptor stimulation. Rather, we provide further evidence that this strategy could efficaciously halt inflammation in IBD.

Of importance, the studies described utilized a compound with an excellent safety profile that is already on the market in some countries, including Italy. Some of the approved drugs used in clinical practice for IBDs, such as mesalazine, cyclosporine, and non-steroidal anti-inflammatory drugs (NSAIDs), have a proven efficacy in symptoms reduction, similarly PDRN was effective in reducing the symptoms and clinical signs, under the experimental conditions described. However, as demonstrated by a recent clinical trial, PDRN is better tolerated and elicits less side effects than common NSAIDs (Squadrito et al., 2014). Despite the fact that no epidemiological data is available regarding the efficacy of PDRN in IBD, it has previously been shown to reduce inflammation in other models (Bitto et al., 2011). To date, none of the previously used experimental drugs that bind to the adenosine receptors have been clinically evaluated. However, since PDRN is currently on the market (for the treatment of poor wound healing), PDRN represents an entity that could be tested in IBD trials. The data presented here strongly suggest that the compound is worthy of study in a human IBD population. In this regard, preliminary pharmacokinetics data obtained in mice posit that PDRN has a half life of approximately 12–17 h (unpublished observations), suggesting it might be suitable for once a day dosing.

In agreement with previous observations, our results demonstrated the beneficial effects of PDRN in two experimental model of colitis. The early onset of the protective effect of PDRN was able to blunt the hemorrhagic diarrhea, improve the loss of weight, and to restore the anatomic integrity of damaged epithelial and mucosal layers. PDRN markedly reduced the inflammatory response and granulocytic infiltration into the mucosal and submucosal layers and, as a consequence, reduced the presence of the circulating pro-inflammatory cytokines TNF-α and IL-1β and reduced MPO activity and lipid peroxidation extent evaluated by MDA in colon samples. PDRN treatment also affected Bax and Bcl-2 expression in experimental colitis reducing apoptotic and necrotic cells in all tissue layers.

In previous experiments PDRN was shown to improve tissue repair (Galeano et al., 2008; Bitto et al., 2011) by reducing inflammatory cytokines and increasing tissue growth factor-beta (TGF-β) and transglutaminase II which are key mediators of tissue remodeling. In a recent clinical trial, Squadrito et al. (2014) confirmed the relevance of these findings by demonstrating that patients with diabetic foot ulcers treated with PDRN had a shorter healing time and lower incidence of wound infections. The same trial also suggested that PDRN efficacy is not related to gender, as the multivariate analysis demonstrated the same rate of healing in both sexes (Squadrito et al., 2014). However, in the present study we have tested PDRN efficacy only in male rats and this can limit the interpretation of these findings to IBD in general.

The mechanism(s) by which PDRN ameliorates experimental colitis are, as yet, undefined though evidence suggests that the effect may be mediated by modulation of inflammatory cell activity. Macrophages are known to have an important role in the induction of tissue injury during colitis and the activation of A_2A_ receptor has a potent macrophage-deactivating effect (Haskò et al., 2000; Kreckler et al., 2006). Activation of the A_2A_ receptor also modulates neutrophil function, regulates the production of reactive oxygen species by these cells (Cronstein et al., 1983, 1985), inhibits the localization of neutrophils to the endothelium by decreasing the expression of the adhesion molecules expressed on neutrophils (Sullivan et al., 2004), and reduces apoptosis of colonic mucosal cells (Mayne et al., 2001).

The A_2A_ antagonist, DMPX, was used to confirm the target of PDRN activity in our experiments and, indeed, abrogated all the therapeutic effects obtained with PDRN. However, recent evidence suggests that DMPX also blocks A_2B_ (Wang et al., 2015). Therefore, it is possible that PDRN could effectively work through other adenosine receptors, including A_2B_. We did not investigate the effects of DMPX alone, which will probably worsen the colitis features, because we wanted to keep to the minimum the number of animals used in this painful procedure, according to the 3Rs principles of animal studies.

## Conclusion

In this study we demonstrated that PDRN, a safe and readily available A_2A_ receptor agonist produced a significant improvement in all the pathological outcomes associated with inflammatory colitis.

## Author Contributions

GaP, AB, and FS designed the study and drafted the paper. GaP, GiP, FG, and NI researched data. GS, SP, GA, GC, AM, and DA critically revised the paper. All authors have approved the final version of the manuscript.

## Conflict of Interest Statement

Author FS has received research support from Mastelli for work on polydeoxyribonucleotide. Authors FS, AB and DA are co-inventors on a patent describing therapeutic polydeoxyribonucleotide activity in chronic intestinal disease. The remaining authors declare that the research was conducted in the absence of any commercial or financial relationships that could be construed as a potential conflict of interest.
