# Corrigendum: Adenosine receptor stimulation by polydeoxyribonucleotide improves tissue repair and symptomology in experimental colitis

**DOI:** 10.3389/fphar.2022.1073445

**Published:** 2022-11-10

**Authors:** Giovanni Pallio, Alessandra Bitto, Gabriele Pizzino, Federica Galfo, Natasha Irrera, Francesco Squadrito, Giovanni Squadrito, Socrate Pallio, Giuseppe P. Anastasi, Giuseppina Cutroneo, Antonio Macrì, Domenica Altavilla

**Affiliations:** ^1^ Section of Pharmacology, Department of Clinical and Experimental Medicine, Medical School, University of Messina, Messina, Italy; ^2^ Department of Human Pathology, University of Messina, Messina, Italy; ^3^ Department of Biomedical Sciences and Morphological and Functional Images, University of Messina, Messina, Italy

**Keywords:** colitis, A_2A_ agonist, PDRN, inflammation, apoptosis

In the published article, the Conflict of Interest statement was incorrectly filed. The correct Conflict of Interest statement appears below.

“Author FS has received research support from Mastelli for work on polydeoxyribonucleotide. Authors FS, AB and DA are co-inventors on a patent describing therapeutic polydeoxyribonucleotide activity in chronic intestinal disease.

The remaining authors declare that the research was conducted in the absence of any commercial or financial relationships that could be construed as a potential conflict of interest.”

The authors apologize for this error and state that this does not change the scientific conclusions of the article in any way. The original article has been updated.

